# Weak Noise in Neurons May Powerfully Inhibit the Generation of Repetitive Spiking but Not Its Propagation

**DOI:** 10.1371/journal.pcbi.1000794

**Published:** 2010-05-27

**Authors:** Henry C. Tuckwell, Jürgen Jost

**Affiliations:** Max Planck Institute for Mathematics in the Sciences, Leipzig, Germany; RIKEN Brain Science Institute, Japan

## Abstract

Many neurons have epochs in which they fire action potentials in an approximately periodic fashion. To see what effects noise of relatively small amplitude has on such repetitive activity we recently examined the response of the Hodgkin-Huxley (HH) space-clamped system to such noise as the mean and variance of the applied current vary, near the bifurcation to periodic firing. This article is concerned with a more realistic neuron model which includes spatial extent. Employing the Hodgkin-Huxley partial differential equation system, the deterministic component of the input current is restricted to a small segment whereas the stochastic component extends over a region which may or may not overlap the deterministic component. For mean values below, near and above the critical values for repetitive spiking, the effects of weak noise of increasing strength is ascertained by simulation. As in the point model, small amplitude noise near the critical value dampens the spiking activity and leads to a minimum as noise level increases. This was the case for both additive noise and conductance-based noise. Uniform noise along the whole neuron is only marginally more effective in silencing the cell than noise which occurs near the region of excitation. In fact it is found that if signal and noise overlap in spatial extent, then weak noise may inhibit spiking. If, however, signal and noise are applied on disjoint intervals, then the noise has no effect on the spiking activity, no matter how large its region of application, though the trajectories are naturally altered slightly by noise. Such effects could not be discerned in a point model and are important for real neuron behavior. Interference with the spike train does nevertheless occur when the noise amplitude is larger, even when noise and signal do not overlap, being due to the instigation of secondary noise-induced wave phenomena rather than switching the system from one attractor (firing regularly) to another (a stable point).

## Author Summary

Many neurons, especially those found in subcortical nuclei, often exhibit repetitive approximately periodic firing of action potentials. We have previously demonstrated how weak noise may inhibit repetitive activity in the Hodgkin-Huxley point model and in pairs of coupled type 1 model neurons. Here we investigated the effects of weak noise in the full Hodgkin-Huxley model which includes a spatial dimension. Our first simulations with noise throughout the whole length of the neuron revealed inhibition of repetitive activity with a minimum as the noise level increased, as in the point model. However, when we reduced the region of application of noise, very surprising results were obtained. Noise right alongside the region of excitation had no effect on the spiking activity. The amount of overlap in space of signal and noise was found to be the key variable in determining whether weak noise would inhibit the firing. If the signal and noise were on disjoint intervals, there was no diminution of activity, no matter how large the spatial extent of the noise. Thus, weak noise that occurred within the signal region could powerfully inhibit spike generation, but such noise immediately outside that region had little effect on the propagation of spikes.

## Introduction

Rhythmic or almost regular periodic neuronal spiking is found in many parts of the central nervous system, including, for example, thalamic relay cells [1]–[3], dopaminergic neurons [4], respiratory neurons [5], [6], locus coeruleus neurons [7] and dorsal raphe serotonergic neurons [7], [8]. Periodic behavior is also found in the activity of neuronal populations [9], [10]. Since stochasticity is a prominent component of neuronal activity at all levels [11], [12], it is of interest to see what effects noise may have on the repetitive activity of neurons. There are many kinds of neuronal model which could be used, an immediate dichotomy being provided by Hodgkin's defining classes of type 1 and type 2 neurons [13], [14]. We have chosen to first examine the behavior of the classic type 2 neural model in its full spatial version [15] which has been employed in recent studies of reliability [16]. The methods we use can be easily extended to more complicated models such as in [1]–[3], [5], [17].

The deterministic spatial Hodgkin-Huxley system, consisting of the cable partial differential equation for membrane voltage and three auxiliary differential equations describing the sodium and potassium conductances is one of the most successful mathematical models in physiology [18]. The corresponding system of ordinary differential equations (ODEs) has been the subject of a very large number of studies and analyses, as for example in references [19]–[28]. Most neuronal modeling studies, aside from some that use software packages, ignore spatial extent altogether and many of those that include spatial extent do not include a soma and hardly ever an axon, because the inclusion of all three major neuronal components, soma, axon and dendrites, makes for a complicated system of equations and boundary conditions. A recent study of spike propagation in myelinated fibres used a multi-compartmental stochastic Hodgkin-Huxley model and demonstrated the facilatory effect of noise and that there were optimal channel densities at nodes for the most efficient signal transmission [29]. In reality, if solutions and statistical properties are found by simulation, stochastic cable models, including the nonlinear model of Hodgkin and Huxley, are not much more complicated than the corresponding point models, although more computing time is required. On the other hand, an apparent disadvantage of spatial models is that more parameters must be specified, many of which can at best only be approximately estimated.

The original HH-system for action potential formation and propagation in squid axon contained only sodium ions, potassium ions and leak currents and the distribution of the corresponding ion channels was assumed to be uniform. That is, the ionic current was
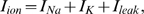
(1)and the various channel densities did not vary with distance. However, there are two reasons why this basic model has been modified in the modeling of more complex cells. Firstly, ion channel densities do depend on position, and secondly, neurons, especially those in the mammalian central nervous system, often receive many thousands of synaptic inputs from many different sources and each source has a different spatial distribution pattern on the soma-dendritic surface [30]–[32]. Thus, spatial models of motoneurons [33] and cortical pyramidal cells [34], [35] have also used the same components for the ionic current as in the HH-system, but with channel densities that vary over the soma-dendritic and axonal surface.

Most central neurons have many dendritic trunks and an axon, each of which branches many times. In this article we focus on a cable model with one space dimension, which is most accurate for a nerve cylinder, usually of uniform diameter. Thus in the first instance our approach is useful to investigate the properties of single axonal or dendritic segments. This simple geometry can nevertheless be used to gain some insight into the properties of neurons with complex anatomy by appealing to such methods as [36] mapping from the neuronal branching structure to a cylinder thus reducing the multi-segment problem to solving a cable equation in one space dimension. Thus single-segment cable models can have relevance for neurons with branching dendritic or axonal trees.

Recent studies of the HH-system of ordinary differential equations (ODEs) with stochastic input have revealed new and interesting phenomena [37], [38] which have a character opposite to that of stochastic resonance [39]. In the latter, there is a noise level at which some response variable achieves a maximum. In particular, in the space-clamped HH system, at mean input current densities near the critical value (about 6.4 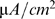) for repetitive firing, it was found that a small amount of noise could strongly inhibit spiking. Furthermore, there occurred, for given mean current densities, a minimum in the firing rate as the noise level increased from zero [37], [38]. Such properties are related to noise-induced delays in firing as found in single HH neurons with periodic input current [40] or networks of such neurons [41], [42]. It is of interest to see if these kinds of phenomena extend to the spatial HH-system where in addition many possibilities for the spatial distribution of the mean input and of the noise. We will demonstrate that the spatial HH system exhibits quite similar but more complex behavior than the ODE system.

## Methods

### The spatial Hodgkin-Huxley model

Based on experimental observations on ionic currents in squid axon, the following system of differential equations was proposed [18] to describe the evolution in time 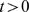 and space 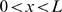 of the depolarization 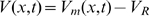, where 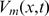 is the actual membrane potential and 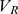 is the (assumed constant) resting potential,
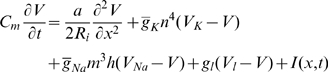
(2)

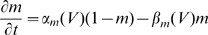
(3)

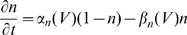
(4)

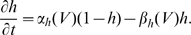
(5)Initial and boundary conditions must of course be specified. The quantities 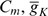, 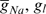, and 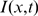 are respectively the membrane capacitance, maximal potassium conductance, maximal sodium conductance, leak conductance and applied current density for unit area (1sq cm). 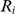 is the intracellular resistivity and 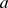 is the fiber radius. The units for these various quantities are as follows: all times are in ms, all voltages are in mV, all conductances per unit area are in mS/cm^2^, 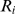 is in ohm-cm, 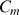 is in 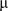
F/cm^2^, distances are in cm, and current density is in microamperes/square cm. 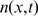, 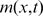 and 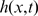 are the dimensionless potassium activation, sodium activation and sodium inactivation variables. Their evolution is determined by the voltage-dependent coefficients
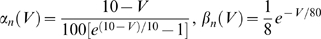


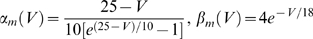


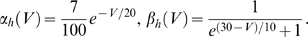
The following standard parameter values are used throughout: 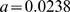, 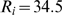, 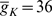, 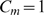, 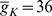, 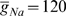, 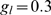, 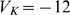, 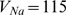 and 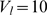. For the initial values, 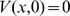, the resting level, and for the auxiliary variables the equilibrium resting values are used, for example
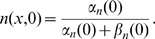
The boundary conditions were chosen to be zero-derivative at both end points.

### Integration technique

The numerical integration of the stochastic HH system of partial differential equations (PDEs) is performed by discretization using an explicit method whose accuracy has been verified by comparison with analytical results in similar systems [43]. For the simulation of stochastic partial differential equations of the type
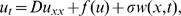
(6)where subscripts denote partial differentiation, 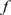 is a given non-random function and 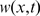 is two-parameter white noise, the following explicit method works well. Suppose the space interval is 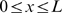 and the time interval is 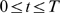. Then put 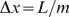 and 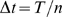 and let 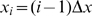 for 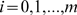, and let 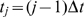 for 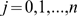. Approximating 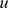 at the grid point 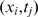 by 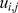 the simulation proceeds by the following Euler scheme:
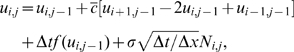
where
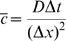
and where the 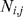
's are a collection of independent standard (zero mean, unit variance) normal random variables. The method generally works well if 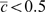 and particularly well if 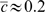. In the present calculations a time step of 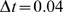 and a space step of 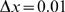 were employed.

## Results

### Deterministic solutions

We firstly consider the HH-system with a deterministic input
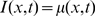
where (see also Figure 3)
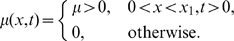
(7)


That is, a constant current is applied indefinitely over a (small) region near the origin. The end-region was chosen for excitation to heuristically represent a soma-dendritic region which is attached to an axon which extends from 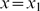 to 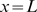. The length was set at 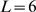.

**Figure 1:**
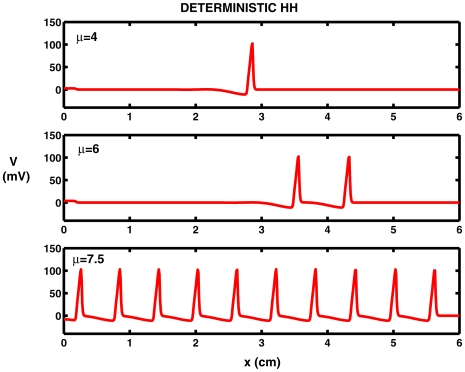
Computed solutions 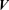 in mV versus distance 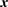 in cm at 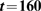 ms of the Hodgkin-Huxley PDE for various current densities 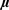 without noise. For remaining parameters, see text.

Examples of the responses are shown in Figure 1. Here, with the stimulus extending to 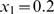 the result for 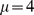 is a solitary spike as seen in the top record of the figure. With 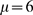, a doublet of spikes propagates along the nerve cylinder as shown in the middle record of the same Figure. Beyond some critical value of 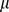 there ensues a train of regularly spaced spikes, as seen with 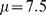 in the bottom record. This response corresponds to repetitive and periodic firing in the HH-system of ODEs. In order to quantify the spiking activity, the maximum number 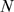 of spikes on 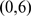 is found.

**Figure 2:**
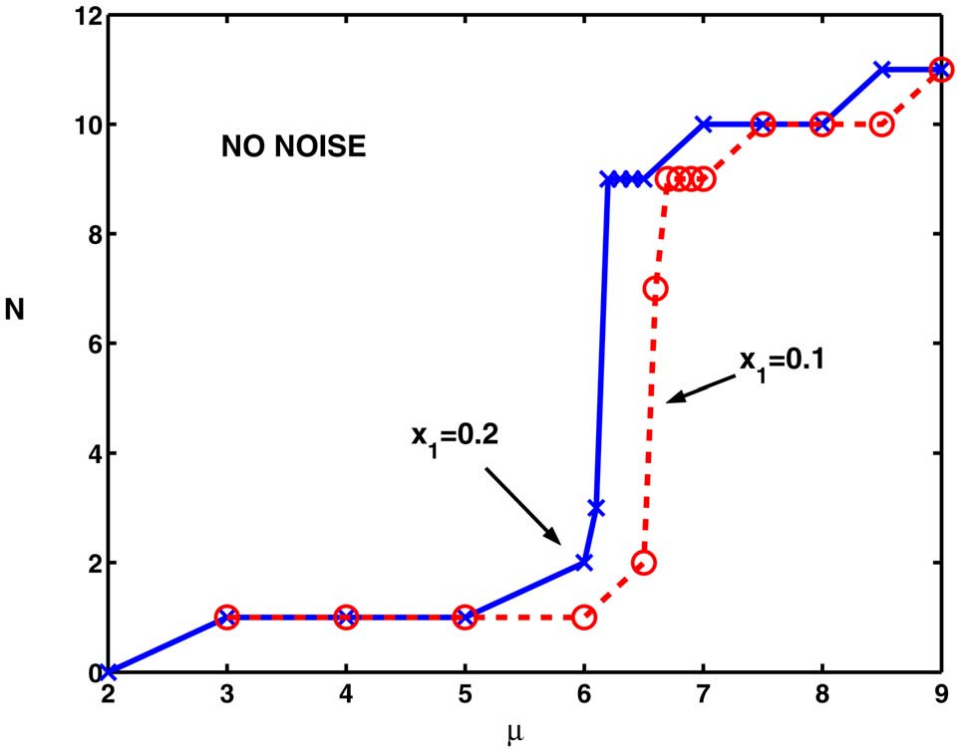
The number of spikes 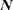 on 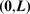 at 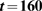 is plotted against the level of excitation 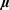 in the absence of noise. The dashed curve is for the smaller region of excitation to 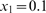 whereas the solid curve is for 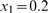. Notice the abrupt increases in spike rates at values close to the birfurcation to repetitive firing, being about 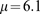 for 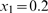 and 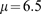 for 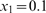.

Figure 2 shows the dependence of 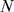 on the (deterministic) input current density, 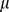, for two values of 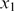, viz 0.1 and 0.2. For 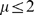 no spikes occurred for both values of 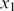. A solitary spike emerged in both cases for 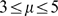 and when 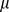 reached 6 in the case of 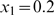 and 6.5 in the case of 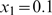, a doublet spike arose and propagated along the cylinder. For slightly greater values of 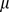, an abrupt increase occurred in the number of spikes, indicating that a bifurcation had occurred, paralleling the appearance of a limit cycle solution in the ODE system. Subsequently, at greater values of 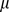, the number of spikes reached a plateau and when 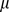 reached 9, the largest value considered here, the number of spikes was 11 for both values of 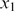. In consideration of the behavior of the HH system of ODEs with noise, it was then of interest to examine the effects of noise on the spike counts near the apparent bifurcation points for the PDE case.

### Excitation with white noise

**Figure 3:**
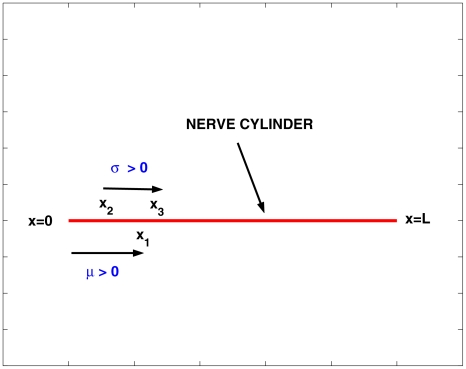
Illustrating the geometry of the set up for stimulation of part or all of a nerve cylinder with white noise. The signal is applied on 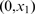 and the noise on 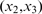.

The HH-system of PDEs was therefore considered with applied currents (consisting of “signal” plus noise) of the following form
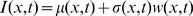
on subsets of a cylindrical nerve cell extending from 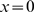 to 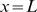. Here 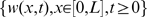 is a two-parameter white noise with covariance function
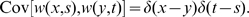
The functions 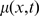 and 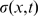 are deterministic and specify the spatial (and temporal) distributions of the mean and variance of the noisy input. The geometrical set-up is illustrated in Figure 3, with 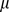 as defined above, so there is mean excitation from the origin to the point 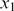. For the random component
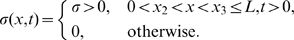
(8)


In order to quantify the spiking activity with noise, we let 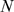 be the (random) number of spikes on 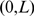 at 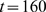 ms, with corresponding mean 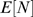. This definition of 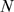 is made because the speed of action potentials with the chosen parameters is such that the first spike generated almost reaches 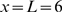 at 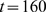 as shown in the bottom record of Figure 1. Figure 2 shows values of 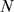 without noise.

### Noise throughout the cable length

**Figure 4:**
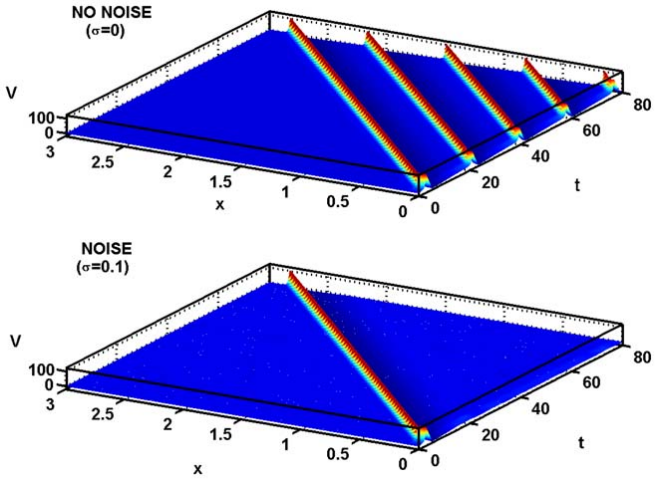
Showing the effects of noise on spiking for mean current densities near the birfurcation to repetitive spiking. Parameters are 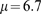, 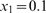, 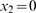, and 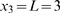. In the top record with no noise there is repetitive firing which, as shown in the bottom record, is strongly inhibited by a relatively small noise of amplitude 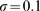.

Here the noise component is 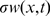 on the whole interval 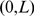. In Figure 4 is shown an example of the effects of noise with the following parameters: 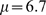, 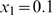, 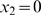, and 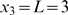. The records show the membrane potential as a function of 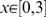 and 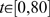. In the top record there is no noise and there are 5 spikes. In the lower record, with a noise level of 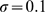 there is a significant diminution of the spiking activity, with only 1 spike. With the noise turned up to 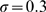 (not shown) the number of spikes is usually greater, but still less than in the noise-free case.

Mean spike counts were obtained with 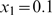, at various 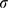 for 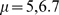 and 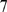. The first of these values is less than the critical value for repetitive firing (see Figure 2) and the other two values are close to and just above the critical value. Relatively small numbers of trials were performed as integration of the PDEs naturally takes much longer than for the ODEs. Hence the number of trials for each point in the following is 50, which is sufficient to show the main effects.

**Figure 5:**
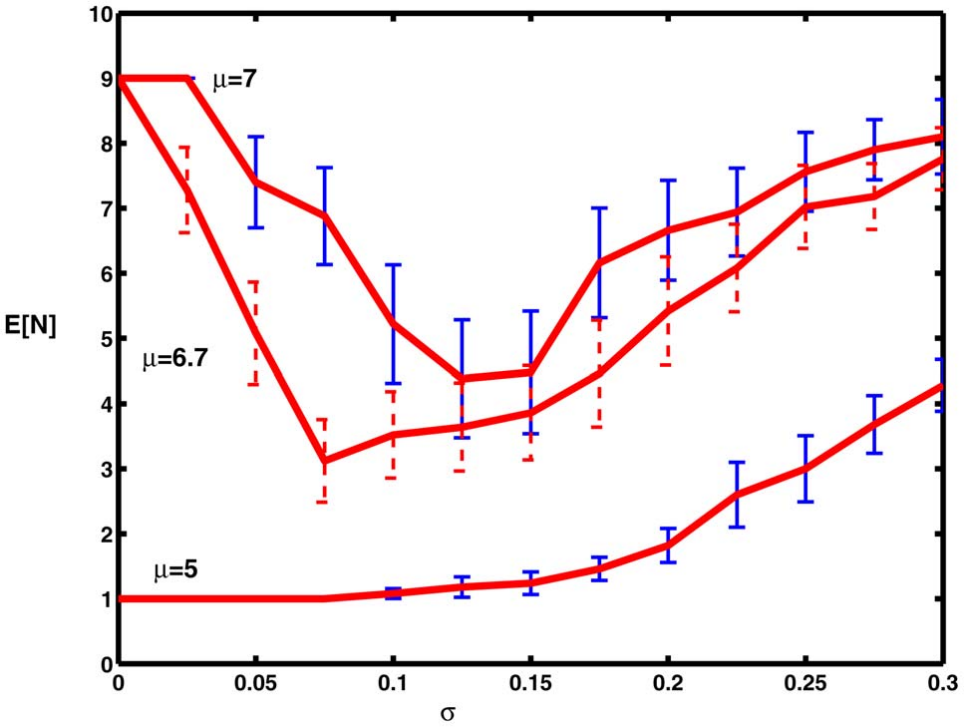
Mean numbers of spikes as a function of noise level for various values of the mean level of excitation 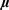 on 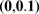. The bottom curve is for a value of 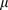 well below the critical value at which repetitive firing occurs. Parameter values 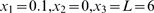.

Figure 5 shows plots of the mean spike counts, 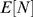, as explained above, versus noise level, along with 95% confidence limits. This figure may be compared with Figure 5 in [38]. For 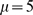, 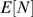 increases monotonically as 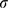 increases from 0 to 0.3. When 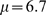, which is very close to the critical value for repetitive firing, a small amount of noise causes a substantial decrease in firing (cf Figure 4) with the appearance of a pronounced minimum near 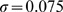. For 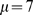, where indefinite repetitive firing occurs without noise, a similar reduction in firing activity occurs for small values of 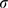, with a minimum near 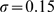, after which spiking activity increases monotonically for values of 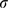 up to the largest value employed, 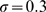. The occurrence of minima with increasing noise level has been referred to as *inverse stochastic resonance* [37].

**Figure 6:**
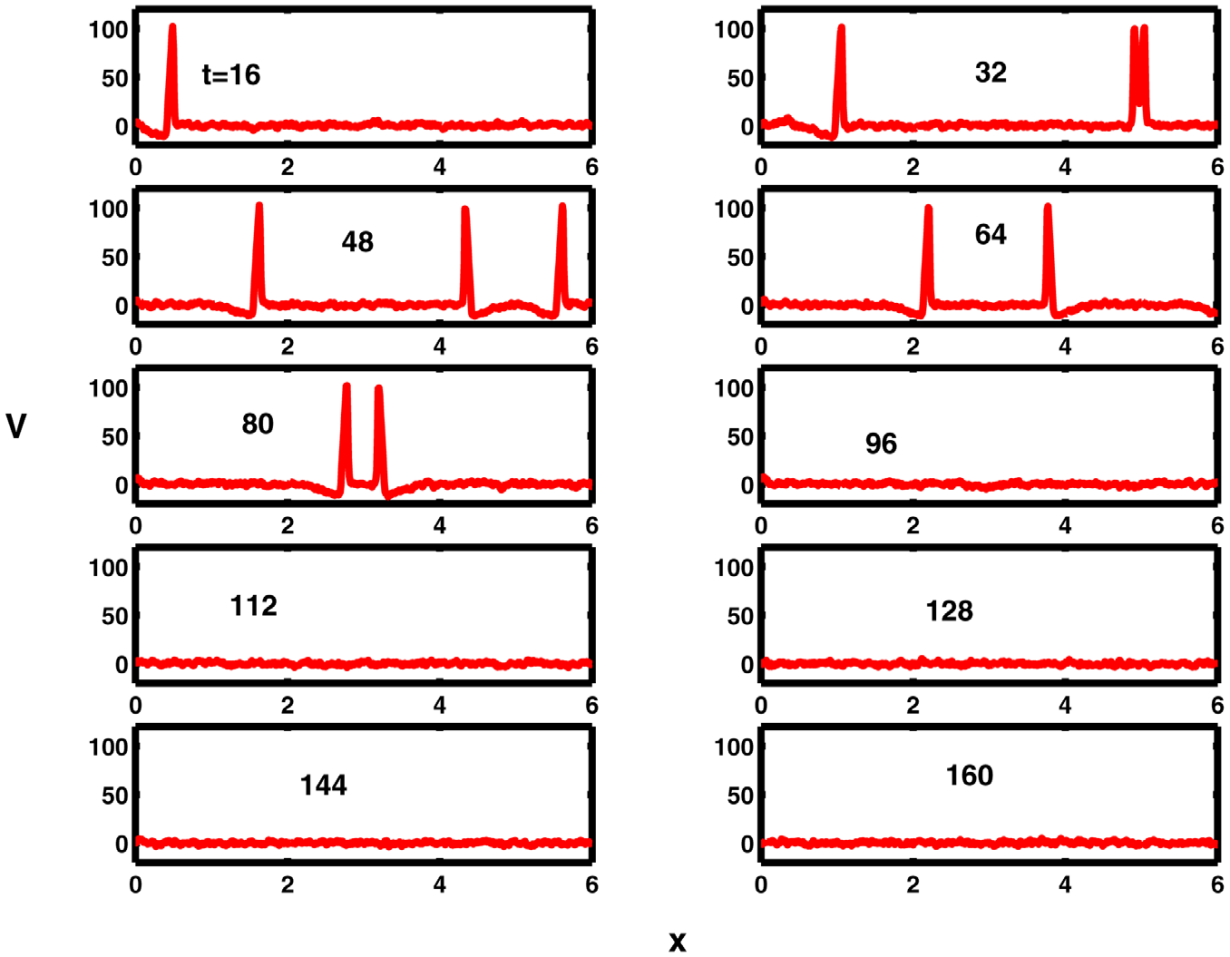
An anomalous case where the spike count on 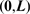 decreases due to secondary waves resulting from large noise. Excitation at the left-hand end gives rise to a solitary action potential seen at 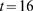. At 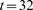 a pair of noise-triggered spikes emerges. The left-going member of the pair collides with the original spike not long after 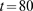 and this pair annihilate one another, leaving no spikes on 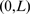 at 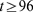. Other parameters 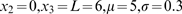.

In some trials, *secondary phenomena* were observed [44] as were also found in the Fitzhugh-Nagumo spatial system, [43]. An example is shown in Figure 6. Here with 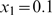, the mean excitation level 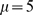 is below the threshold for repetitive firing and noise of amplitude 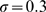 is applied along the whole cable. A single spike emerges from the left hand end as seen at 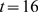. By 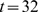 a pair of spikes is seen to emerge at 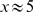, one traveling towards the emerging spike and one to the right. Not long after 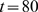 the left-going secondary spike collides with the emerging right-going spike and these spikes annihilate each other. Thus, the spike count on 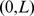 ends up at 0 at 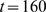 due to inteference between the left-going noise-generated spike and the right-going spike elicited by the excitation applied on 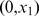.

**Figure 7:**
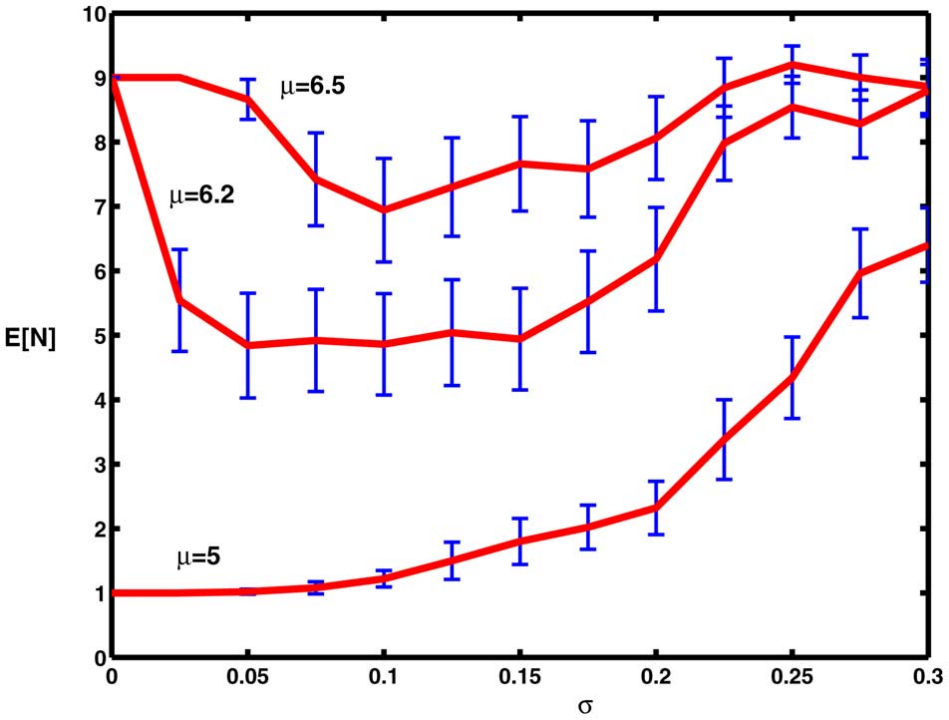
Mean numbers of spikes as a function of noise level for various values of the mean level of excitation 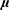 on 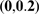. The bottom curve is for a value of 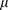 well below the critical value at which repetitive firing occurs. Other parameters 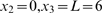.

With a larger region of excitation, so that 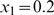, mean spike counts were similarly obtained with various noise amplitudes for values of 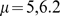 and 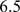. Again, the first of these values is less than the critical value for repetitive firing (see Figure 2) and the other two close to or just above the critical value. Inspection of Figure 7 shows that the behavior is similar to that for 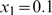. These findings parallel those found for the HH system of ODEs and although there is no standard bifurcation analysis for the PDE system, it is probable that most of the arguments which apply to the system of ODEs apply in some sense to the PDEs (see Section 5).

**Figure 8:**
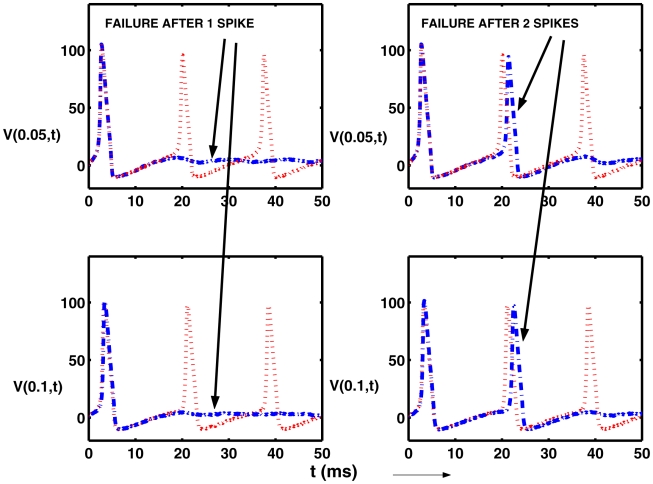
Voltage trajectories illustrating weak noise induced failure of the emergence of a repetitive train of action potentials. Upper plots show 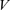 as a function of time at 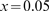 and the lower plots show 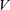 at at 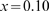. In all 4 subplots are shown trials in red (dotted) in which no failure occurred. In the left panels failure is manifest as the occurrence of only one action potential (blue dash-dot curves) whereas in the right hand example there are two spikes preceding failure (blue dash-dot curves). Parameters are 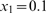, 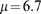, 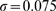, 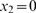 and 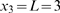.

In order to explore the mode of spike failure, we examined the early behavior of the voltage near the source of action potentials. This was done with 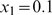, 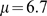, 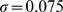, 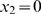 and 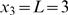. With these parameters there is a high failure rate as can be seen in Figure 5. Consider then the trajectories shown in Figure 8. The upper sets are the potential versus time at 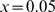 and the lower sets are for 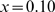, at the edge of the signal region. In all subplots are shown in red (dots) spike trains with no failed spikes despite the noise. In the left panels, the blue (dash-dot) curves are for a trial in which only one spike emerged. In the right panels, the blue (dash-dot) curves are for a trajectory with only two spikes. (The choice of markings is to enable almost coincident spikes to be distinguished.) It can be seen that voltage paths in cases of failure are close to those for the repetitive spike train until just before the 2nd or 3rd etc spike is about to form, whereupon the trajectory wanders on a path away from threshold. Consequently, the spike train terminates prematurely as the system thereafter stays at low levels of depolarization, destroying the possibility of further spikes. Evidently, there is a very small probability of a noise-driven passage to the spiking regime after the trajectory is driven off it for these relatively small values of 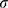, though of course if 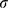 were much larger, noise-induced spiking would occur within a short time. It is noteworthy that (a), there were never trajectories with zero spikes, which was also the case in the ODE case; and (b), there was not, in 50 cases examined, a single instance in which there was a spike at 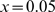 and not at 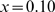; that is, failure, if it occurred, did so at smaller values of 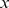 within the signal region. The reason for this last observation will need further investigation of the effects of spatially distributed sources.

### Noise on small intervals

**Figure 9:**
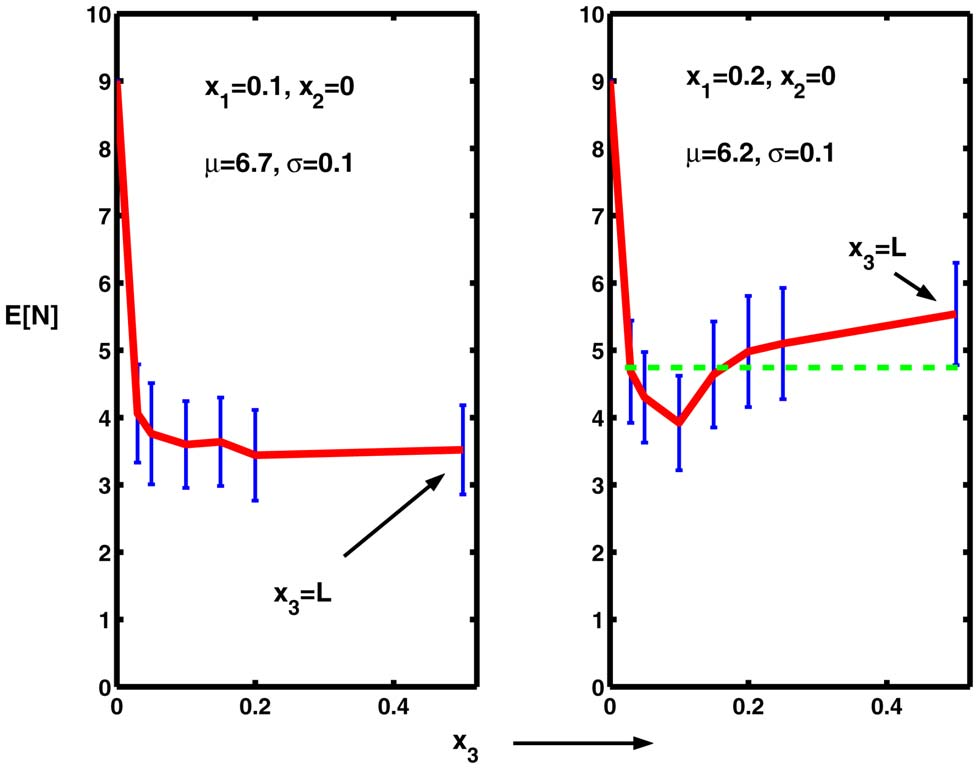
Mean numbers of spikes with noise, at level 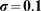, restricted to small regions, from 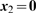 to 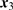, that overlap partially or completely the small interval 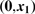 at the left-hand end at which the neuron receives excitation with mean 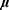 **.** In the left graph, 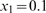 and 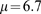 whereas in the right graph 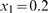 and 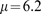. The point 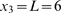 is included in each case (not to scale).

In the model we are considering, the relatively small region 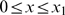 where 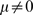 is akin to the input (somatic) region of the neuron and the segment 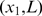 corresponds to the axon. In the previous subsection noise was present throughout the whole interval 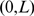. It is of interest to see how varying the extent of the noise around the somatic region affects the propagation of action potentials. Thus the noise was limited to 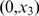 with 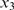 taking values from near zero to 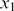 and somewhat greater. The same two values of 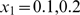 as above were employed, so there was partial or complete overlap of the region with noise and the region with excitatory input. For both values of 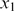, the value of 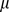 was chosen to be just at the critical values for repetitive spiking, being 6.7 for 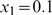 and 6.2 for 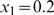. The results for the mean spike counts (50 trials) are shown in Figure 9 along with 95% confidence limits.

For 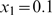, as shown in the left part of Figure 9, noise over even the very small region to 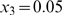 reduces the mean spike count by 48% and when the extent of the noise is slightly greater the mean spike count drops to about 40% of its value without noise. On the same graph is given the result for noise on the whole interval and it can be seen that there is no statistically significant (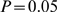) reduction in spiking compared to 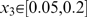. In the right hand part of the same figure, similar results are shown for 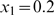. Generally the same remarks apply as for 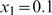 but it can be seen from the dashed line from the lower confidence limit for 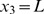 that there is a possibly significant difference between the spike counts for 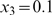 and 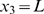, but no difference for any other pair of 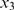-values. Thus the apparent minimum in the mean count for the case 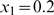 is probably not statistically significant, although this matter could profit from a further more detailed analysis. The results of Figure 9 show that weak noise over even a small region where the signal occurs may inhibit partially or completely the emergence of spikes just as or almost as effectively as weak noise along the whole extent of the neuron. This is not the case for noise of sufficient strength to induce spiking outside the signal region.

### Contrasting overlap and non-overlap of noise and signal

It was at first surprising that, with 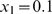, 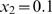 and 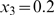, when there was weak noise just to the right of the excitatory stimulus, no reduction in spike count occurred. Thus, it seemed that weak noise at the source of the spiking could cause a significant reduction in spike count, but noise with the same magnitude and extent over a region disjoint from the region of excitation, tended to have little or no effect on spike propagation. To investigate this further, the excitatory signal was applied with strength 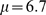 on 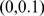 and noise of amplitudes 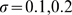 and 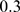 was activated on the (disjoint) regions from 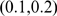, 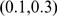 and 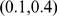, which are of successively increasing lengths. On no occasion for any of these disjoint intervals or for any noise level, was there a reduction in spiking activity, although as expected, the timing of the spikes was slightly different in each case. Furthermore, when the signal was applied on 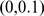 and the noise (at all three levels) was applied on 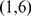, there was still no reduction in spiking activity even though the spikes encountered noise on about 84% of distance along the cable.

**Figure 10:**
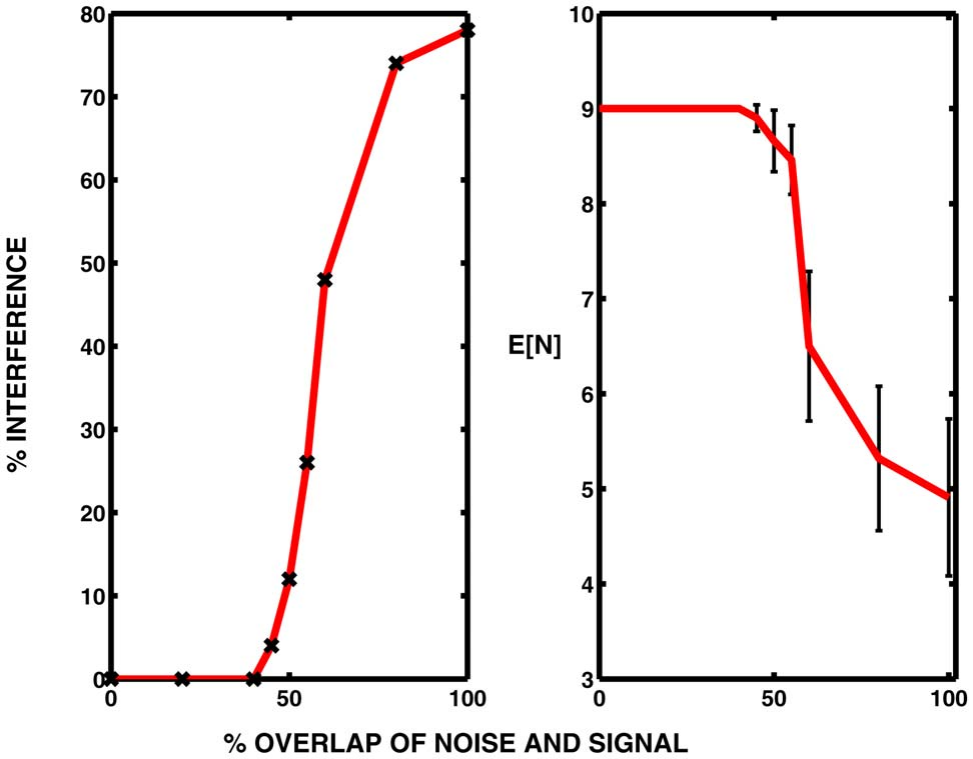
Interference of spike trains by noise. *Left panel*. The fraction of trials during which weak noise interfered with the spike train is plotted against the % overlap of the regions where the signal 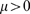 and the noise amplitude 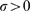. Parameter values are 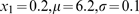. *Right panel*. The corresponding expected number of spikes 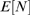 is plotted against % overlap of signal and noise. 95% confidence limits are shown based on 50 trials.

In a systematic investigation, with the mean excitation fixed at 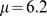 for 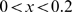, noise of strength 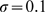 was applied for 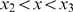 where 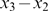 was fixed at 0.2 and 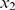 varied from 0, corresponding to complete overlap, to 0.2, corresponding to zero overlap. The results, which are shown in Figure 10, provide a clear demonstration of the significance of the degree of overlap of (weak) noise and signal. Histograms of spike counts on 50 trials enabled the determination of the fraction of trials on which there was interference of the spike train by noise. For example, with complete overlap (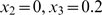) there were 11 of 50 trials with a full complement of 9 spikes as in the noise-free case, representing interference, mainly in the form of inhibition, by noise in 78% of trials. In contrast, with 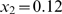 and 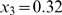, giving 40% overlap, there were 9 spikes in all 50 trials, indicating zero interference. The probability of interference (as a %) versus degree of overlap is plotted in the left panel of Figure 10. This probability is seen to remain at zero until the overlap is 40% and then increases monotonically to achieve the value near 80% when the overlap is complete. In the right panel of Figure 10 are plotted the mean spike counts versus amount of overlap along with 95% confidence intervals. The mean spike count remains at 9 until the overlap is greater than 40%. Note that if the mean spike count is 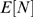 and there are 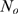 spikes without noise, then the left panel plots 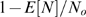 as a %.

The results of Figure 10 illustrate dramatically the importance of overlap of signal and weak noise for the latter to have an inhibitory effect on spiking. That is, a spike will generally pass through a region of weak noise, but if the same noise is applied at the source of the spike, there is a considerable chance of the non-emergence of one or more spikes.

Finally, it was decided to see if having the location of excitation near the end point 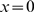 was playing a role in the above effects. With no noise, there were at 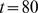, 4 spikes on either side of the stimulus (with 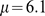 there was only 1). For noise of amplitude 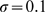, with the region of application of the noise overlapping exactly the stimulus region, there was a reduction in spiking activity by over 30%, there being several instances with only 1 spike. On the other hand, when the signal and noise regions were disjoint, the latter being 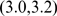, there was no reduction in spiking activity.

### The spatial HH system with conductance-based noise

The above results were obtained with additive noise, but in neurons, noisy input that has synaptic origin is better approximated via random processes that describe more accurately the properties of synaptic transmission [45], [46]. In a simplified model of synaptic input the current density 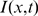 in (2) is replaced by
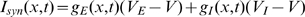
(9)where 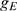 and 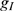 are the excitatory and inhibitory conductance densities and 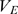 and 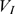 are the reversal potentials for excitatory and inhibitory synaptic currents. Using diffusion approximations for 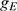 and 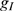 we have the stochastic partial differential equations
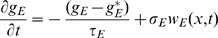
(10)

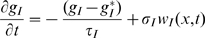
(11)where the 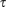
's are time constants of decay, 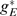 and 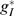 are equilibrium conductance values, 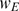 and 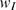 are independent standard spatio-temporal white noises and 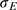 and 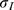 are noise amplitudes.

An investigation of the many possible spatial configurations of excitatory and inhibitory synaptic inputs is outside the scope of the present article. However, in order to demonstrate that the phenomenon of inhibition by weak noise extended to the spatial Hodgkin-Huxley model with conductance-based noise, an excitatory synaptic input was employed whose spatial distribution paralleled that in an additive noise case. Thus with 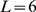 and 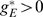 only for 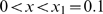, repetitive spiking was found to occur without noise when 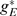 was greater than a threshold value a little less than 0.112. Three values of 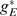 were then employed; a just greater than threshold value of 0.112, a value well above threshold at 0.13, and a value well below threshold at 0.08. For these values of 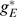, simulations were performed for various 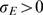 on 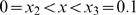; that is, with noise and signal overlapping completely on a small interval near the origin. The remaining parameter values were 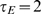 ms and 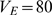 mV relative to resting potential. As for the additive noise case, the (random) number 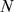 of spikes was recorded on the whole cable length at time 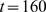 ms, by which time the first emitted spike has almost reached the end 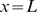.

**Figure 11:**
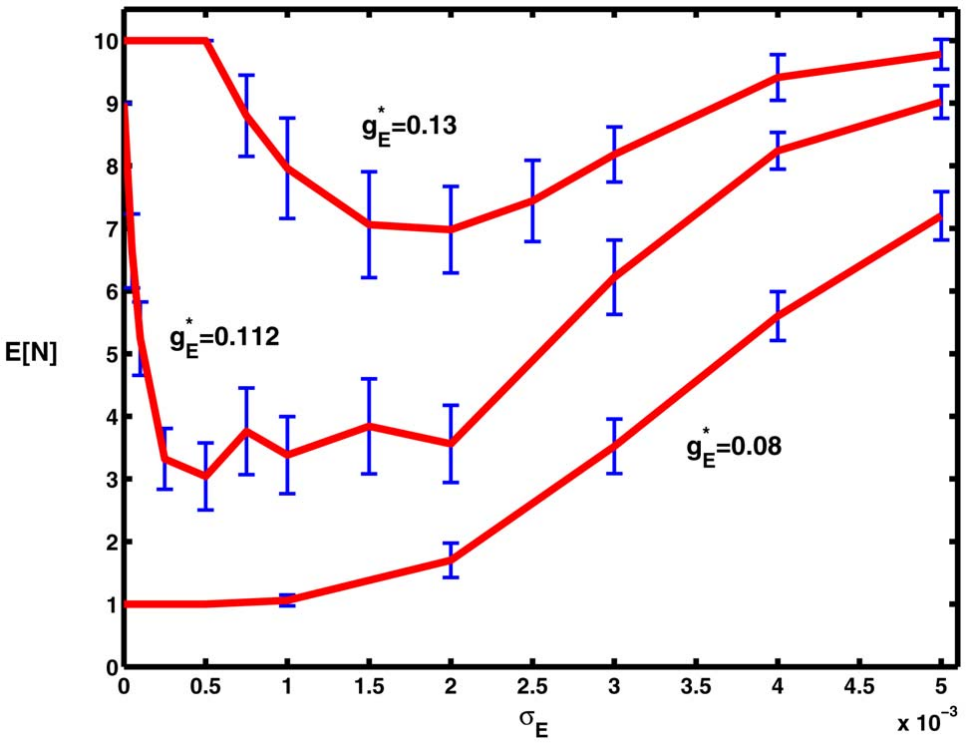
Mean numbers of spikes for the HH system with conductance-based input as a function of noise level for three values of the mean level of excitation 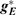 on 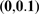. The top, middle and bottom curves are for values of 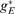 well above, just above and well below the critical value for repetitive firing. The indicated 95% confidence intervals are based on 50 trials at each data point. Parameter values 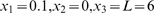.

Results for the expected number, 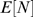 of spikes are plotted against noise amplitude 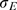, along with 95% confidence intervals in Figure 11.

For the uppermost curve, above the critical value of 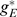, there are 10 spikes with no noise and very small noise. As the noise increases the mean number of spikes drops to a minimum of about 7 when 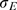 is near 0.002 and then increases back to about 10 when 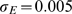. With the value of 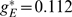 just above the critical value (middle curve), 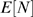 decreases rapidly from 9 to about 3.5 as the noise increases from zero, but the minimum is rather diffuse. At the smallest (subthreshold) value of 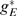, there is only a monotonically increasing number of spikes from 1 about 7 to as the noise amplitude increases from 0 to 0.005. Thus, the effects of weak noise on repetitive spiking in the spatial HH system in the conductance-based case parallel those for additive noise for the spatial configuration considered. A more complete study will be reserved for future work.

## Discussion

As pointed out in the Introduction, the effects of noise in the point-model (ODE) version of the Hodgkin-Huxley neuron have been considered in many articles, but only a few have considered the stochastic spatial version of this neural model. In the ODE model, noise applied near the critical mean input current density for repetitive firing led, at small amplitudes, to a substantial reduction in spiking activity and an interesting minimum in the firing rate as the noise level increased away from zero. The inhibitory effect of noise on spiking has been experimentally demonstrated in the squid axon [47]. Such an inhibitory effect has been explained in transitions from one attractor, a limit cycle, to another, being a stable rest point [28], [38].

It was natural, therefore, to see if such effects induced by noise in the ODE system also arose in the HH system of PDEs. In the present article we have found that similar phenomena do in fact occur in the HH PDE (cable) system, as can be seen particularly in Figures 5 and 7. With (deterministic) excitation at the left-hand end of the cable, when relatively weak additive uniform two-parameter white noise is applied, there is indeed an inhibition of the spiking activity and furthermore, a minimum occurs as the noise strength increases for signal strengths near the critical value for repetitive spiking. This was also the case for conductance-based (synaptic) input. However, there are two new effects in the spatial HH system that cannot arise in the ODE system and which clearly demonstrates the utility of spatial models as providing more realistic insights into the behavior of real neurons, which do of course have considerable, and sometimes very large, spatial extent.

Firstly, the spatial distributions of the signal and the noise may not be the same. Investigation of this aspect revealed unsuspected properties. The main finding was that if the noise and signal overlapped completely or partially, then weak noise could inhibit the firing activity. However, if signal and noise were on disjoint intervals, then weak noise had no effect. This was the case even when the noise extended along the major part of the cable. Heuristically, one could argue that weak noise can prevent the generation of action potentials (at their source) but not their propagation. It will be of much interest to explore the mathematical reasons for this behavior in more detail.

Secondly, in spatial models (or real neurons), secondary effects may be induced by noise if it is sufficiently strong. For example, noise may itself lead to the generation of (usually pairs of) action potentials at locations which are possibly remote from the regions of application of a signal. This was seen in Figure 6 for the HH system and previously in the Fitzhugh-Nagumo system [43]. Noise induced action potentials can sometimes just be spurious or they can annihilate previously generated spikes which they encounter.

The above results are intriguing and to us were unexpected. They suggest that in the HH system the inhibition of spiking by noise of small amplitudes (here for 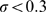) is only significant if the region of signal generation and the region of occurrence of noise overlap, possibly only to a minor degree. That noise and other spurious stimuli have an effect on (neuronal) pacemakers is well documented [47]–[50]. In central neurons the geometry and neurophysiology are much more complex than that considered in this article. If rhythmic spiking is instigated in a locally noisy environment it is feasible that there may be noise-induced failure to spike. However, rhythmic spikes which arise in dendritic regions and then propagate to the soma through noisy regions will probably not be inhibited by weak noise. Future work on these complex phenomena involving noise is needed for the elucidation of their role, not just in the relatively simple HH model, but in more realistic models of central nervous system neurons such as [1]–[6]. We anticipate that the kind of results we have obtained here extend in essence to models which describe in detail the anatomy of the soma, dendrites and axon.

### Mathematical background

For getting mathematical insight into the phenomena just described, we should distinguish two different regimes in Equations (2)–(5). We have the small region 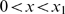 where an external current is applied and where consequently the spikes are generated, and the large region 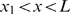 where no such current is applied and where the spike is propagated. The first region was found to be much more sensitive to perturbations than the second.

The spatial Hodgkin-Huxley equations belong to the class of reaction-diffusion systems, and some general theory can be applied, see e.g. [51], [52]. The typical nonlinear effects are generated by the interaction between the nonlinear reaction term and the linear diffusion term. In the first regime, where the spike is generated, the reaction dominates the behavior. Therefore, the effects of perturbations are similar to those in the non-spatial Hodgkin-Huxley equations which constitute a system of nonlinear ordinary differential equations. In particular, noise when applied at a particular part of the periodic trajectory that corresponds to the regular spiking can destroy an incipient spike, see [38].

The second regime is modelled as a traveling wave solution of the Hodgkin-Huxley equations, see [53]. Here, a traveling wave is a solution of the above PDE system that depends only 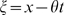. With 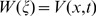 and 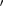 denoting a derivative with respect to 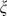, on introducing the auxiliary function 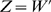, we obtain the first order system 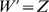 and
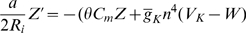


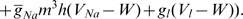
The changes for the remaining equations are obvious. The existence of traveling waves for such systems has been investigated in [54]. The difference with the ordinary Hodgkin-Huxley equation consists in the term 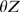 on the right hand side. According to the analysis of [55], this has the consequence that the fast reaction dynamics corresponding to the propagated spike branches off from the vicinity of the equilibrium set 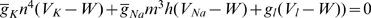 at positions that are different from the original rest state 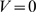. Therefore, the region at the incipient spike where the solution slowly traverses a narrow region of its basin of attraction, as analyzed in [28], is avoided. Consequently, the traveling wave is much less sensitive to perturbations than the spike generation. This yields a qualitative explanation of our numerical findings.
